# Granulin, a novel STAT3-interacting protein, enhances STAT3 transcriptional function and correlates with poorer prognosis in breast cancer

**DOI:** 10.18632/genesandcancer.58

**Published:** 2015-03

**Authors:** Jennifer E. Yeh, Simion Kreimer, Sarah R. Walker, Megan M. Emori, Hannah Krystal, Andrea Richardson, Alexander R. Ivanov, David A. Frank

**Affiliations:** ^1^ Department of Medical Oncology, Dana-Farber Cancer Institute, Boston, MA; ^2^ Barnett Institute of Chemical and Biological Analysis, Northeastern University, Boston, MA; ^3^ Department of Medicine, Brigham and Women's Hospital and Harvard Medical School, Boston, MA; ^4^ Department of Pathology, Brigham and Women's Hospital and Harvard Medical School, Boston, MA

**Keywords:** STAT3, interacting protein, breast cancer

## Abstract

Since the neoplastic phenotype of a cell is largely driven by aberrant gene expression patterns, increasing attention has been focused on transcription factors that regulate critical mediators of tumorigenesis such as signal transducer and activator of transcription 3 (STAT3). As proteins that interact with STAT3 may be key in addressing how STAT3 contributes to cancer pathogenesis, we took a proteomics approach to identify novel STAT3-interacting proteins. We performed mass spectrometry-based profiling of STAT3-containing complexes from breast cancer cells that have constitutively active STAT3 and are dependent on STAT3 function for survival. We identified granulin (GRN) as a novel STAT3-interacting protein that was necessary for both constitutive and maximal leukemia inhibitory factor (LIF)induced STAT3 transcriptional activity. GRN enhanced STAT3 DNA binding and also increased the time-integrated amount of LIF-induced STAT3 activation in breast cancer cells. Furthermore, silencing GRN neutralized STAT3-mediated tumorigenic phenotypes including viability, clonogenesis, and migratory capacity. In primary breast cancer samples, GRN mRNA levels were positively correlated with STAT3 gene expression signatures and with reduced patient survival. These studies identify GRN as a functionally important STAT3-interacting protein that may serve as an important prognostic biomarker and potential therapeutic target in breast cancer.

## INTRODUCTION

Breast cancer is the most frequently diagnosed cancer and second leading cause of cancer death in American women, accounting for about 230,000 new cases and 40,000 deaths per year [1]. Triple-negative breast cancers (TNBCs), which do not express estrogen receptor or progesterone receptor, or overexpress HER2, are a particularly aggressive subtype of breast cancer characterized by high rates of metastases and poor prognosis [2]. TNBCs also represent a major cancer health disparity as they occur more frequently and with higher mortality rates in young African-American and Hispanic women [3].

Since cancer is associated with aberrant gene expression patterns, increasing attention has been focused on transcription factors like signal transducer and activator of transcription 3 (STAT3) which lies at the convergence points of many oncogenic signaling pathways. STAT3 is one of a family of transcription factors that mainly reside in the cytoplasm until activated by phosphorylation on a conserved tyrosine residue 705 (PY-STAT3) by growthfactor receptor tyrosine kinases, cytokine-receptorassociated Janus kinases (JAKs), or non-receptor tyrosine kinases [4]. Although unphosphorylated STAT3 (U-STAT3) can also form dimers and shuttle into the nucleus [5, 6], tyrosine phosphorylation enhances STAT3 dimerization and translocation into the nucleus where it regulates genes involved in cell proliferation, differentiation, survival, and angiogenesis. In normal cells, STAT3 activation is transient due to tight regulation by inhibitory molecules [4]. However, cancer cells, including TNBC cells, frequently have inappropriate constitutive activation of STAT3, and can be dependent on STAT3 activity for survival [7]. Besides tyrosine phosphorylation, STAT3 can undergo a number of other post-translational modifications including serine phosphorylation (PSSTAT3) [8-10], acetylation [11], and methylation [12]. Aside from its involvement in transcriptional regulation, STAT3 has also been reported to play a role in the mitochondrial electron transport chain [9, 10] and microtubule dynamics [13, 14].

STAT3 constitutes an important therapeutic target because cancer cells depend on the inappropriate activity of this oncogenic transcription factor while normal cells can often tolerate disruption of these proteins with little toxicity due to redundancies in normal signaling pathways [4]. However, unlike kinases which have well-defined ATP binding pockets in which small molecules can be designed to fit, transcription factors have large surface areas for protein-DNA and protein-protein interactions which are more difficult to target [15]. One strategy to inhibit oncogenic transcription factors like STAT3 is to identify critical cofactors that modulate their function.

Several proteins have been reported to interact with STAT3 and modify its function, including STAT3- interacting protein 1 (StIP1) which preferentially associates with unphosphorylated STAT3 to promote STAT3 interaction with upstream kinases [16], transcriptional cofactors [17, 18], and proteins involved in STAT3 cellular trafficking into the nucleus [19] or the mitochondria [8, 20]. However, proteins that functionally interact with STAT3 in breast cancer have yet to be defined. Here we report the use of a proteomics approach to identify a novel STAT3-interacting protein that modulates STAT3 activity and may play an important role in the pathophysiology of breast cancer.

## RESULTS

### STAT3 interacts with GRN in breast cancer cells

**Fig. 1:**
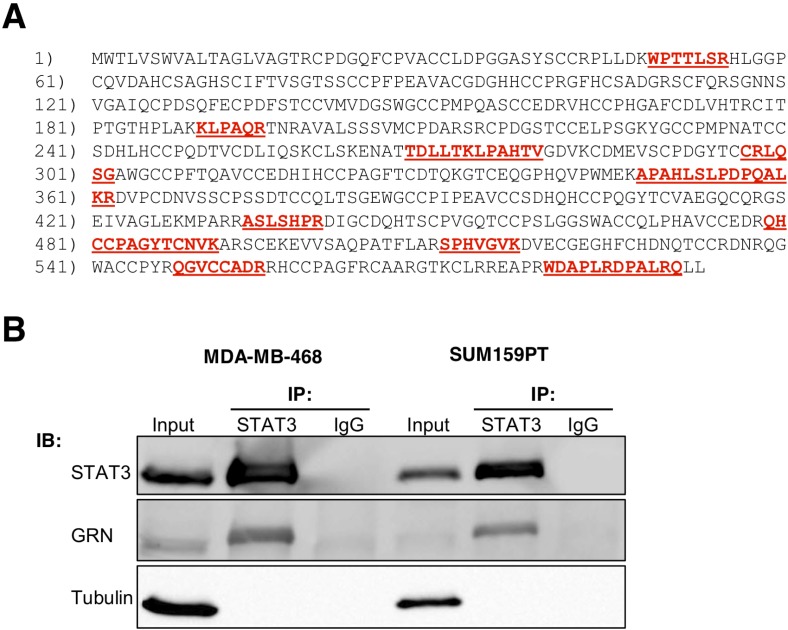
STAT3 interacts with GRN in breast cancer cells (A) Mass spectrometry coverage of GRN amino acid sequence P28799. Peptides identified by PEAKS or Proteome Discoverer are indicated in red underline. (B) STAT3-GRN interaction in MDA-MB-468 and SUM159PT cells was analyzed by immunoprecipitation using antibodies against STAT3 (or non-specific immunoglobulin G, IgG) followed by immunoblots with the indicated antibodies. Input indicates 5% of pre-immunoprecipitated samples.

To identify novel STAT3-interacting proteins that modulate STAT3 activity, we utilized the TNBC cell lines MDA-MB-468 and SUM159PT, which display constitutively active STAT3 signaling including tyrosine phosphorylation of STAT3 at the 705 residue (PY-STAT3), and are dependent on STAT3 function for growth and survival [7]. We performed immunoprecipitations (IP) of STAT3 followed by liquid chromatography tandem mass spectrometry analysis (LC-MS/MS) shotgun proteomic profiling. Two hundred and eighty-five protein groups were identified by at least 6 peptide-spectrum matches (PSMs) and at least 2 unique peptide sequences in STAT3-containing complexes. Among these, granulin (GRN) was detected by 10 unique peptides corresponding to 15.5% sequence coverage spanning the entire amino STAT3. We first validated the STAT3-GRN interaction acid sequence of the protein (Fig. 1A). Because GRN in breast cancer cells by immunoprecipitating STAT3 was detected in two independent experimental replicates and immunoblotting with a GRN antibody (Fig. 1B). We from two breast cancer cell lines with constitutively confirmed this STAT3-GRN interaction by reciprocal coactive STAT3, it was selected for further analysis. In immunoprecipitation (co-IP) in TNBC cells transiently addition, GRN was not previously known to interact with expressing GRN (Supp. Fig. S1). We also performed immunofluorescence assays and found that GRN colocalized with STAT3 in the nucleus of TNBC cells (Supp. Fig. S2).

### GRN enhances constitutive STAT3 transcriptional activity

**Fig. 2:**
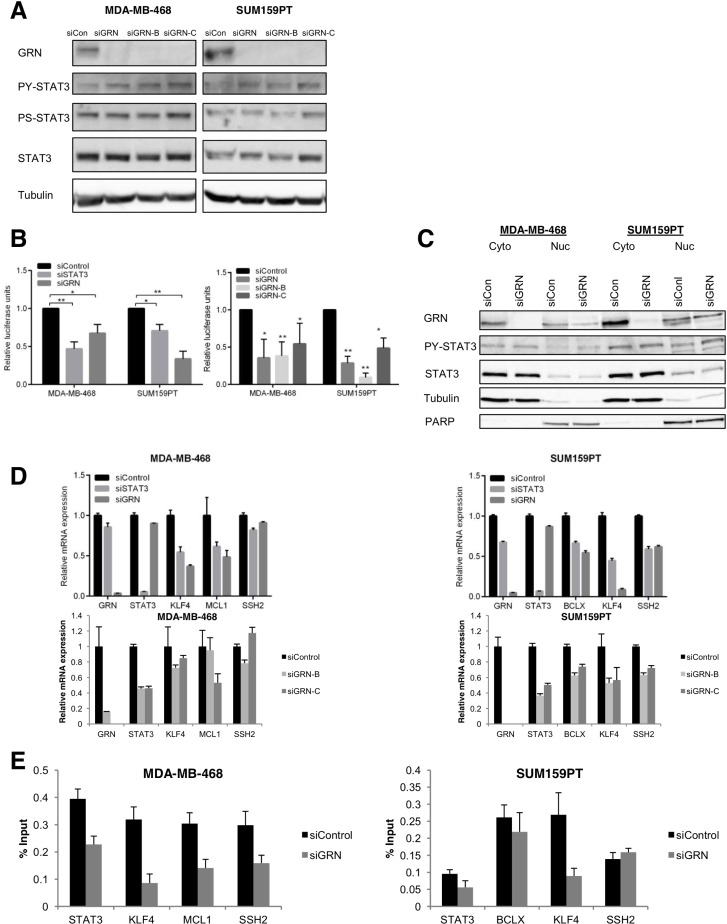
GRN is necessary for constitutive STAT3 transcriptional activity in triple-negative breast cancer cells MDA-MB-468 and SUM159PT cells were transfected with siRNA targeting GRN (pool siGRN and individual constructs siGRN-B and siGRN-C) or a non-targeting control (siCon). They were then analyzed by (A) immunoblot for levels of phosphorylated and total STAT3 in whole cell lysates, (B) luciferase reporter assay for STAT3-dependent transcriptional activity (N = 3), (C) nuclear and cytoplasmic fractions (tubulin serves as a loading control for the cytoplasmic fraction and PARP serves as a loading control for the nuclear fraction), (D) qRT-PCR for expression of endogenous STAT3 target genes (normalized to GAPDH; N = 3), and (E) chromatin immunoprecipitation with an antibody for STAT3 followed by PCR at the indicated STAT3 target genes (representative of N = 3).

Given the identification of a STAT3-GRN interaction in TNBC cells that have constitutively active PY-STAT3, we hypothesized that GRN positively regulates STAT3 activity in TNBC cells. To investigate the effect of GRN on STAT3 activity in TNBC cells, we silenced GRN in MDA-MB-468 and SUM159PT cells using small interfering RNA (siRNA). We tested the effect of two individual constructs, siGRN-B and siGRN-C, as well as an siRNA pool, siGRN, that includes both constructs. Both individual siRNA constructs showed similar activity as the pool in reducing GRN protein levels (Fig. 2A). However, silencing GRN did not significantly alter STAT3 tyrosine phosphorylation (PY-STAT3) or serine phosphorylation (PS-STAT3).

We then determined whether GRN knockdown affects STAT3 transcriptional activity by transfecting cells with a luciferase reporter gene under the control of a STAT3 transcriptional response element. This reporter construct specifically measures STAT3-mediated transcriptional activity in these TNBC cells, which do not have constitutive activation of other STAT family members [7]. Silencing GRN reduced STAT3-dependent luciferase activity by 40-60%, similar to the effect of silencing STAT3 which was used as a positive control for reducing STAT3 transcriptional activity (Fig. 2B). siGRN-B and siGRN-C showed similar activity as the pool siGRN in reducing STAT3 transcriptional activity by at least 50% (Fig. 2B). To verify that this reduction of STAT3 activity was specifically mediated by GRN knockdown, we performed a rescue experiment by expressing mouse GRN, which is not predicted to be recognized by the siRNA constructs targeting human GRN based on sequence analysis. Although cotransfection of mGRN with siGRN was not able to fully restore physiological levels of GRN, it partially rescued the effect of silencing GRN on STAT3 transcriptional activity, suggesting that the effect of GRN depletion on STAT3 transcriptional function is an on-target effect (Supp. Fig. S3).

To understand the mechanism by which GRN facilitates STAT3 transcriptional activity, we considered the possibility that GRN affects the nuclear localization of activated STAT3 in TNBC cells. To test this hypothesis, we isolated nuclear and cytoplasmic fractions from TNBC cells transfected with siRNA targeting GRN. Consistent with our immunofluorescence results, GRN was detected in both nuclear and cytoplasmic fractions of these cells. However, silencing GRN did not reduce the nuclear levels of PY-STAT3 (Fig. 2C).

We next considered the possibility that GRN affects STAT3 binding to cognate genomic sites. We examined a subset of STAT3-regulated genes based on published STAT3 gene expression signatures [21-23] and STAT3 ChIP-Seq data we had generated. We then validated that these genes are direct STAT3 targets, defined as showing at least a 30% reduction in STAT3 DNA binding upon STAT3 knockdown (Supp. Fig. S4). GRN knockdown reduced the mRNA expression of these genes, similar to the effects of STAT3 knockdown (Fig. 2D). Then we performed chromatin immunoprecipitation (ChIP) with an antibody specific for STAT3 in TNBC cells with GRN knockdown or control. Because the individual siRNA constructs showed similar effects as the pool on depleting GRN protein levels and reducing STAT3 transcriptional activity in the aforementioned experiments, the siRNA pool (siGRN) was used for ChIP. Of the three STAT3 target genes examined in MDA-MB-468 cells, there was on average a 58% decrease in STAT3 association at these sites following GRN knockdown (Fig. 2E). Of the three STAT3 target genes examined in SUM159PT cells, one (*KLF4*) showed a reduction in STAT3 association upon GRN knockdown while the other two (*BCLX* and *SSH2*) did not, even though GRN knockdown reduced the mRNA levels of these genes by about 50%. Taken together, these results suggest that GRN may modulate the expression of STAT3 target genes in multiple ways. For some STAT3 target genes, GRN enhances STAT3 DNA binding. For other genes, GRN does not significantly affect STAT3 DNA binding but may play a role in recruiting critical transcriptional cofactors instead.

### GRN is necessary for maximal cytokine-induced STAT3 transcriptional activity

**Fig. 3:**
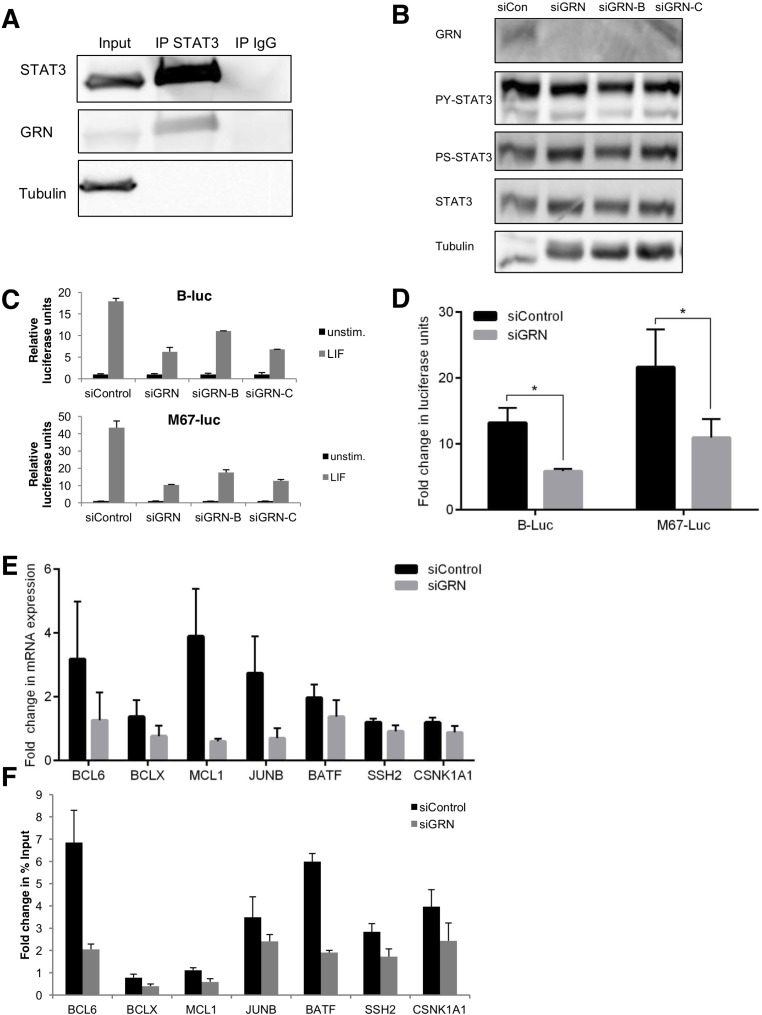
GRN is necessary for maximal cytokine-induced STAT3 transcriptional activity in breast cancercells (A) STAT3-GRN interaction in SK-BR-3 cells was analyzed by immunoprecipitation using antibodies against STAT3 (or non-specific IgG) followed by immunoblot with the indicated antibodies. Input indicates 5% of pre-immunoprecipitated samples. SK-BR-3 cells were transfected with siRNA targeting GRN (pool siGRN and individual constructs siGRN-B and siGRN-C) or a non-targeting control (siCon), then stimulated with LIF were analyzed by (B) immunoblot and (C) luciferase reporter assay for STAT3-dependent transcriptional activity. SK-BR-3 cells transfected with siRNA targeting GRN (pool) were analyzed by (D) luciferase reporter assay for STAT3-dependent transcriptional activity (B-luc, M67-luc) (N = 3), (E) qRT-PCR for expression of endogenous STAT3 target genes (normalized to HPRT; N = 6), and (F) chromatin immunoprecipitation with an antibody to STAT3 followed by PCR at the indicated STAT3 target genes (data normalized to unstimulated cells; representative of N = 2).

To determine if an association between GRN and STAT3 could be found in breast cancer cells that do not have constitutively active PY-STAT3, we examined SKBR-3 cells, a HER2-overexpressing breast cancer cell line in which STAT3 can be specifically activated by cytokine stimulation. We found that STAT3 and GRN could be coimmunoprecipitated in these cells, even in the absence of stimulation (Fig. 3A). To investigate the role of GRN on cytokine-induced STAT3 activity, we silenced GRN with siRNA and then stimulated with leukemia inhibitory factor (LIF). We again compared the effect of two individual siRNA constructs to an siRNA pool and found that the individual siRNAs showed similar effects as the pool in not reducing STAT3 tyrosine or serine phosphorylation while suppressing the LIF-induced activity of two STAT3dependent luciferase reporters (Fig. 3D). Because the individual siRNA constructs showed similar effects as the pool on depleting GRN protein levels and reducing STAT3 transcriptional activity, the siRNA pool (siGRN) was used for subsequent experiments.

In addition to LIF, STAT3 activation can also be induced by other cytokines that signal through glycoprotein 130 (gp130), including interleukin-6 (IL-6) and oncostatin activation (Supp. Fig. S5B). We chose LIF for subsequent M (OSM) (Supp. Fig. S5A). Silencing GRN also reduced experiments because it induces robust stimulation of IL-6 and OSM-induced STAT3 transcriptional activity as STAT3 tyrosine phosphorylation and is physiologically measured by luciferase reporter assay, indicating that this relevant in breast cancer cell proliferation [24, 25]. These effect of GRN is not dependent on the stimulus for STAT3 luciferase reporter constructs can respond to STAT5 and STAT1 activity as well as STAT3 activity; however, LIF does not induce STAT5 tyrosine phosphorylation in these SK-BR-3 cells (Supp. Fig. S6A). Since cytokines that signal through gp130 can also activate STAT1, we next evaluated the effect of granulin on STAT1-dependent transcription. We transfected SK-BR-3 cells with siRNA targeting GRN (both with a pool and two individual constructs), then stimulated with IFNγ, which specifically induces STAT1 but not STAT3 tyrosine phosphorylation (Supp. Fig. S6A). We analyzed both luciferase reporter activity as well as expression of established STAT1 target genes [26]. Silencing GRN had no significant effect on STAT1 tyrosine phosphorylation, STAT1-dependent transcriptional activity, or STAT1 gene expression (Supp. Fig. S6B-D). Thus, changes in luciferase expression with GRN silencing in these systems reflect its effect on STAT3-dependent transcription.

Since a STAT3-dependent luciferase reporter provides a global measure of STAT3 activity, we next examined specific endogenous genes known to be regulated by STAT3 and found that silencing GRN suppressed the induction of these genes (Fig. 3E). We next considered potential mechanisms by which GRN reduced cytokine-induced STAT3 transcriptional activity. Based on previous STAT3 ChIP data in TNBC cells, we hypothesized that GRN modulates STAT3 binding to DNA. To determine whether GRN decreases LIF-mediated STAT3 recruitment to cognate binding sites, we performed ChIP with an antibody to STAT3. Of the genes with greater than 1.5-fold induction of STAT3 binding in control cells, there was on average a 36% decrease in STAT3 association following GRN knockdown (Fig. 3F).

Given previous reports identifying GRN upregulation in breast tumor-instigating cells, we next examined the effects of extracellular GRN on STAT3 function in breast cancer cells. Recombinant human progranulin (PGRN) did not induce STAT3 tyrosine phosphorylation nor did it enhance the phosphorylation of STAT3 induced by LIF (Supp. Fig. S7A). In addition, PGRN had no effect on STAT3-dependent luciferase activity in SK-BR-3 cells (Supp. Fig. S7B). To determine whether extracellular PGRN had effects on STAT3 activity in tissue types besides breast cancer, we examined a STAT3-dependent luciferase reporter cell line derived from sarcoma cells [27]. We found that silencing GRN reduced IL-6 induced STAT3 transcriptional activity in these cells, suggesting that GRN is necessary for maximal STAT3 function in this cell system as well (Supplemental Fig. S7C). However, extracellular PGRN did not induce STAT3 transcriptional activity, nor did it further increase IL-6-induced STAT3 transcriptional activity in these cells (Supplemental Fig. S7D).

Since we found that GRN enhances the transcriptional activity of constitutively phosphorylated STAT3 in TNBC cells, we considered whether extracellular PGRN could further increase STAT3 activity in these cells. However, treatment with PGRN did not significantly alter STAT3 tyrosine phosphorylation (Supp. Fig. S8A) or STAT3 target gene expression (Supp. Fig. S8B) in TNBC cells. Taken together, these findings indicate that while intracellular GRN contributes to STAT3-dependent gene transcription, extracellular PGRN has no significant effect on STAT3 function.

### GRN increases the kinetics of STAT3 phosphorylation and nuclear translocation

**Fig. 4:**
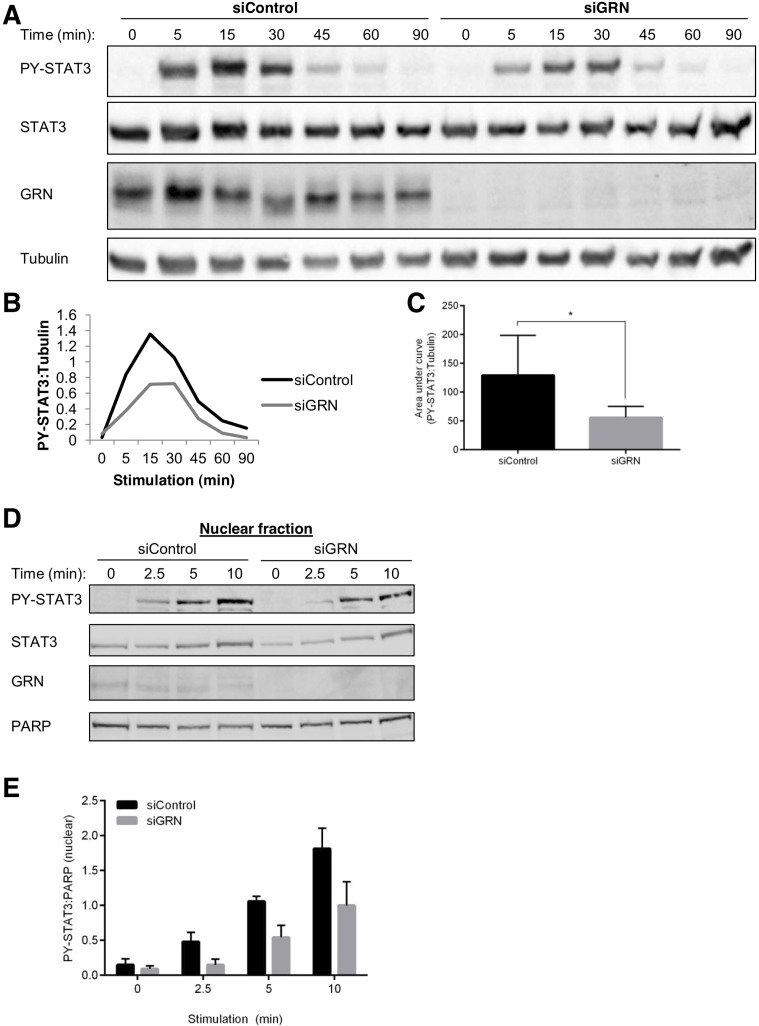
GRN enhances the kinetics of cytokine-stimulated STAT3 activation and nuclear translocation (A) SK-BR-3 cells were transfected with siRNA targeting GRN or a non-targeting control, then stimulated with LIF for the indicated times. Cells were then analyzed by immunoblot for levels of phosphorylated and total STAT3 in whole cell extracts. Tubulin serves as a loading control. (B) Immunoblot band intensities plotted as ratios of phosphorylated STAT3 to tubulin. (C) Area under the curve of phospho-STAT3:tubulin (N = 5). (D) SK-BR-3 cells were transfected with siRNA targeting GRN or a non-targeting control, then stimulated with LIF for the indicated times. Cells were then analyzed by immunoblot for levels of phosphorylated and total STAT3 in nuclear fractions. PARP serves as a nuclear fraction loading control. (E) Immunoblot band intensities plotted as ratios of phospho-STAT3 to PARP (N = 4).

Given the decrease in LIF-induced STAT3 genomic binding following GRN depletion, we considered the possibility that GRN modulates the kinetics of LIF-induced STAT3 tyrosine phosphorylation. To test this hypothesis, we analyzed whole cell lysates from SK-BR-3 cells transfected with siRNA targeting GRN or a non-targeting control then stimulated with LIF for times ranging from 0-90 minutes (Fig. 4A). We normalized immunoblot band intensities for PY-STAT3 to a loading control protein, tubulin, and plotted these values versus time. Silencing GRN decreased the slope and the maximum of LIF-induced STAT3 tyrosine phosphorylation (Fig. 4B), and also reduced the area under the curve (AUC^time^) of this normalized STAT3 tyrosine phosphorylation over time (Fig. 4C), indicating less overall STAT3 activation. These findings are consistent with the observed reductions in STAT3 DNA binding and gene expression.

To determine whether these reductions in STAT3 activation extend to nuclear trafficking of STAT3, we isolated nuclear fractions from SK-BR-3 cells transfected with siRNA targeting GRN and stimulated with LIF. Silencing GRN delayed the nuclear accumulation of PYSTAT3 (Fig. 4D). Immunoblot band intensities for PYSTAT3 normalized to a nuclear fraction loading control, PARP, were reduced following GRN knockdown (Fig. 4E). Over the entire timecourse of LIF-induced STAT3 tyrosine phosphorylation and dephosphorylation, silencing GRN reduced the AUC^time^ of nuclear normalized PYSTAT3 (Supp. Fig. S9), indicating less cumulative PYSTAT3 in the nucleus. These findings indicate that GRN enhances cytokine-induced STAT3 activation and nuclear accumulation.

### GRN enhances the tumorigenic phenotype of TNBC cells

**Fig. 5:**
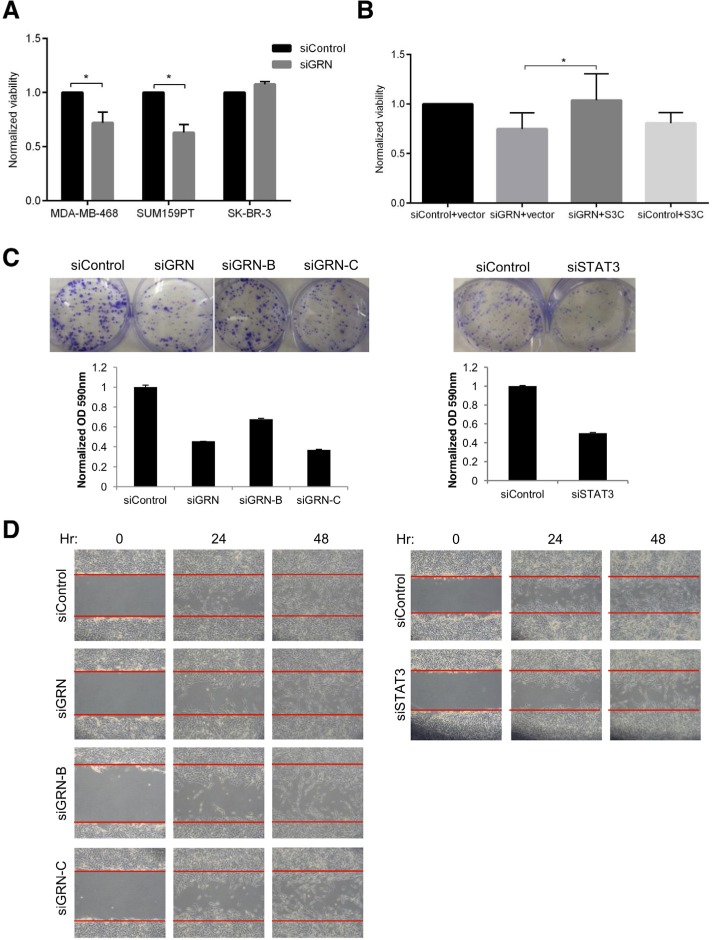
GRN enhances TNBC cell tumorigenic phenotypes (A) Viable cell number was quantitated by ATP-dependent assay for MDAMB-468, SUM159PT, and SK-BR-3 cells transfected with siRNA targeting GRN (N ≥ 3). (B) Viable cell number was quantitated forSUM159PT cells transfected with siRNA targeting GRN (or control) and an expression construct for constitutively active STAT3 (S3C)or vector control (N = 2). SUM159PT cells transfected with siRNA targeting GRN (pool siGRN and individual constructs, siGRN-B andsiGRN-C) or STAT3 were analyzed by (C) clonogenicity and (D) wound healing assays.

Given the necessity of GRN for maximal STAT3 transcriptional activity and the critical role of STAT3 in breast tumorigenesis, these studies raise the possibility that GRN may be a potential therapeutic target in TNBC cells, which have inappropriate activation of STAT3. To test this hypothesis, we determined the effect of depleting GRN by RNA interference on the phenotype of these cells. Silencing GRN reduced the viability of MDA-MB-468 and SUM159PT cells by 30% but did not reduce the viability of SK-BR-3 cells, suggesting that inhibiting GRN preferentially affects cells with activated STAT3 (Fig. 5A). To determine whether the effect of GRN on cell viability is mediated through a STAT3-specific pathway, we utilized a constitutively active form of STAT3 which can activate transcription of STAT3 target genes without exogenous cytokine stimulation [28]. Cotransfection of STAT3C with siRNA targeting GRN attenuated the effect of GRN knockdown on reducing STAT3 target gene expression (Supp. Fig. S10A&B) and reversed the effect of GRN knockdown on SUM159PT viability (Fig. 5B). These findings suggest that the effect of granulin depletion is mediated by decreased STAT3 transcriptional activity.

To determine if silencing GRN affects other STAT3-mediated tumorigenic phenotypes, we performed clonogenesis and migration assays. Silencing GRN decreased the clonogenesis of SUM159PT cells by approximately 70%, similar to the magnitude of depleting STAT3 (Fig. 5C). Silencing GRN also reduced the motility of SUM159PT cells to a comparable extent as silencing STAT3 (Fig. 5D). Interestingly, STAT3C did not attenuate the effect of GRN knockdown on SUM159PT migration (Supp. Fig. S10C), suggesting that some but not all phenotypes associated with GRN knockdown can be rescued by constitutively active STAT3.

### GRN expression correlates with STAT3 gene expression signatures and reduced patient survival in human breast cancers

**Fig. 6:**
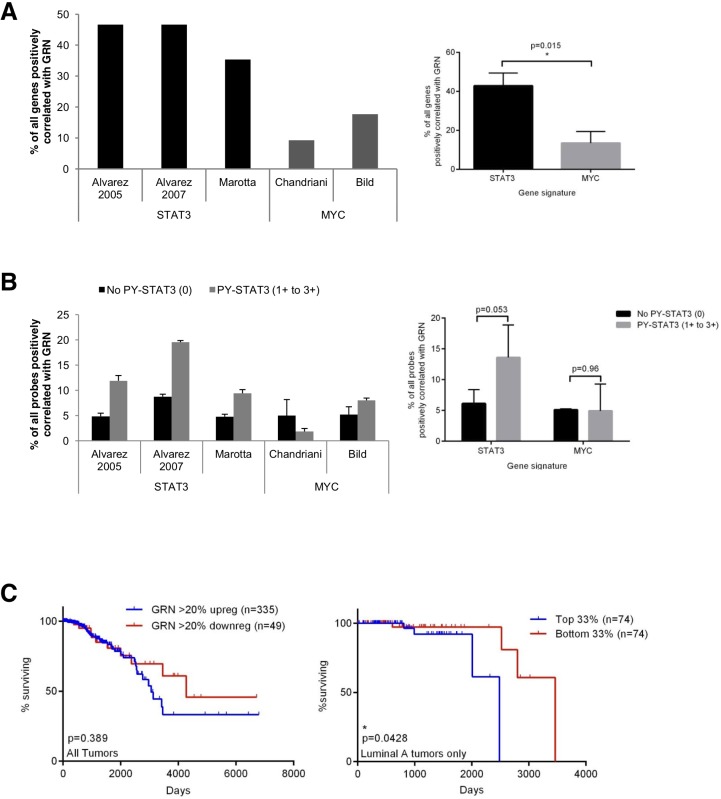
GRN correlates with STAT3-dependent gene expression and poor prognosis in breast cancer patients (A) Pearson correlation of GRN mRNA expression with STAT3 and MYC signature genes in breast tumors from The Cancer Genome Atlas. T-test was used to compare the percent of positively correlated genes in STAT3 signatures vs. MYC signatures. (B) Pearson correlation of GRN mRNA expression with STAT3 and MYC signature genes in breast tumors segregated by nuclear STAT3 tyrosine phosphorylation. Data are means ± SD of analysis with 3 probes to GRN. T-test was used to compare the percent of positively correlated probes for tumors with and without nuclear staining for STAT3 tyrosine phosphorylation. (C) Kaplan-Meier survival curves (log-rank test) for TCGA cohort of all (left) and luminal A (right) breast tumors.

Given that GRN is necessary for maximal STAT3dependent gene expression in breast cancer cells *in vitro*, we considered whether GRN expression is also correlated with STAT3-dependent gene expression *in vivo*. We analyzed gene expression data from 529 breast cancers (The Cancer Genome Atlas; TCGA). Of these, 64% displayed increased GRN expression whereas only 9% displayed decreased GRN expression, with a cutoff of 20% change compared to a universal human reference RNA (Stratagene) derived from cell lines representing different human tissues. We then analyzed the genome-wide linear correlation between mRNA expression of GRN and individual genes in previously defined STAT3 gene expression signatures derived from murine fibroblasts [21], primary human breast cancers with histological evidence of STAT3 tyrosine phosphorylation [22], and HS578T human basal-like breast cancer cells [7]. As a control, we performed correlation analysis of GRN expression with genes in signatures for the unrelated oncogenic transcription factor MYC [29, 30]. GRN was positively correlated with an average of 42.9% of genes in STAT3 signatures but with an average of only 13.5% of genes in MYC signatures (Fig. 6A), supporting the preferential association of GRN expression with signatures indicative of functional STAT3 activation in primary breast tumors.

GRN enhances the transcriptional activity of tyrosine-phosphorylated STAT3, and the knockdown of GRN preferentially reduces the viability of breast cancer cells with constitutively active PY-STAT3. Therefore, we considered whether the association between GRN expression and a STAT3-dependent gene expression signature would be more pronounced in breast cancers displaying STAT3 tyrosine phosphorylation. We utilized a gene expression dataset of 64 breast tumors in which we had previously performed immunohistochemistry to nuclear PY-STAT3 [31]. As expected given our findings that silencing GRN did not reduce PY-STAT3 levels in TNBC cells, there was no correlation between GRN expression and PY-STAT3 in these tumors, although there was a correlation between GRN expression and tumor grade (Supp. Fig. S11).

The correlation between GRN expression and STAT3 signature genes was increased by an average of 2.2-fold in tumors with evidence of STAT3 tyrosine phosphorylation (PY-STAT3 scores of 1+ to 3+) compared to tumors that lacked STAT3 tyrosine phosphorylation (PY-STAT3 scores of 0). In contrast, the correlation between GRN expression and MYC signature genes did not significantly change in tumors grouped by STAT3 tyrosine phosphorylation (Fig. 6B). These findings indicate that in primary breast cancers, GRN expression specifically correlates with enhanced STAT3 transcriptional activity in the presence of tyrosine-phosphorylated STAT3, consistent with our *in vitro* findings.

To investigate the clinical relevance of GRN expression, we performed Kaplan-Meier survival analysis on patients in the TCGA breast cancer data set for whom both gene expression and clinical outcome was available. Increased GRN expression was associated with reduced overall survival in all breast cancer patients (Fig. 6C), a trend that was statistically significant for patients with luminal A tumors, which express estrogen receptor and/or progesterone receptor but do not overexpress HER2. Luminal A tumors also express activated STAT3, albeit at lower frequency compared to TNBC tumors [7]. Survival analysis in triple-negative tumors was not performed due to the limited number of TNBC tumors in the dataset (12%), which mirrors the lower percentage of this subtype in the overall patient population.

## DISCUSSION

STAT3, a convergence point of oncogenic signaling, is inappropriately activated in clinically aggressive TNBC tumors which have limited therapeutic options. To identify STAT3-interacting proteins that contribute to STAT3 tumorigenesis, we performed comprehensive profiling of proteins interacting with STAT3 in TNBC cells which have constitutively active STAT3. This study revealed GRN as a novel STAT3 interactor that is necessary for constitutive as well as maximal cytokine-induced STAT3 transcriptional activity in breast cancer cells.

GRN is produced as an 88 kDa glycoprotein that is proteolytically cleaved into 6 kDa peptides called granulins [32]. GRN is an autocrine growth factor [33-35] that has been reported to bind to the intracellular protein positive transcription elongation factor (P-TEFb), altering its subcellular distribution to repress the transcription of tumor suppressor genes [36]. In addition to its growth-promoting function, GRN is also an established survival factor in cancer models. GRN expression in breast cancer patient tissue and sera is positively correlated with the more aggressive triple-negative subtype of breast cancer; associated with resistance to the breast cancer drugs tamoxifen, trastuzumab, and doxorubicin; and associated with an increased risk of tumor recurrence [33, 37-39]. These findings suggest a critical role for GRN not just in promoting growth but also in bestowing tumor cells with aggressive phenotypic features. Finally, GRN has demonstrated effects on the tumor microenvironment. GRN-expressing bone marrow cells induce stromal fibroblasts to express genes involved in inflammation and matrix remodeling, thereby promoting cancer metastasis [39].

In TNBC cells which have constitutively activated STAT3, silencing GRN reduced STAT3 binding to a subset of STAT3 target genes, mirroring the effect of silencing GRN on the mRNA expression of these genes. Depleting GRN protein did not reduce the levels of constitutive STAT3 activation as measured by phosphorylation of a critical tyrosine residue (Y705) in these TNBC cells. However, silencing GRN did suppress the time-integrated amount of cytokine-induced PY-STAT3 in whole cell and nuclear fractions of SK-BR-3 cells. Given the effects of GRN knockdown on the cumulative amount of cytokineinduced PY-STAT3 in this system, one possible mechanism is that the STAT3-GRN interaction may be initiated in the cytoplasm and subsequently affect downstream gene regulation in the nucleus by helping to recruit RNA polymerase II or transcriptional coactivators. Indeed, we detected the presence of GRN in both cytoplasmic and nuclear fractions of breast cancer cells, and silencing GRN reduced STAT3 DNA binding and target gene expression in SK-BR-3 cells. The differential effect of silencing GRN on PY-STAT3 in breast cancer cells with constitutive versus cytokine-inducible STAT3 activation suggests that GRN may have multiple collaborating mechanisms of modulating STAT3 activity including affecting the dynamics of cytokine-stimulated STAT3 phosphorylation and also modifying the DNA binding of STAT3 or other transcriptional cofactors.

Our findings that silencing GRN mirrors the effect of silencing STAT3 on reducing TNBC cell viability, clonogenesis, and migration suggest that inhibiting GRN may neutralize STAT3-mediated oncogenic behavior. Moreover, analysis of gene expression data in breast cancer patients revealed a significant correlation between GRN expression and STAT3 gene signatures, increased tumor grade (Fig. S6A), and reduced patient survival. Thus, targeting STAT3 in combination with its cofactor GRN may represent a viable and novel approach for treating aggressive tumors like TNBC.

One example of STAT cofactors that have been targeted in cancer therapy is the bromodomain family of proteins, which bind to acetylated histones and modulate the epigenetic environment to promote transcription factor activity. A small-molecule bromodomain inhibitor, JQ1, decreased STAT5-dependent transcriptional activity in leukemia cells with constitutive STAT5 activation [40]. Moreover, JQ1 provided a synergistic effect with existing tyrosine kinase inhibitors for treating STAT5driven malignancies. JQ1 has also been reported to reduce STAT5 function and disrupt the maturation of dendritic cells, suggesting a beneficial effect of this drug in treating inflammatory-driven diseases [41]. These findings demonstrate the therapeutic potential of targeting transcriptional coactivators.

In conclusion, we have used a proteomics approach to identify GRN as a novel STAT3-interacting protein that enhances STAT3 transcriptional activity and may play an important role in the pathophysiology of breast cancer. These findings provide insight into STAT3-dependent gene regulation and function and suggest a new approach to developing transcription factor-based cancer therapies.

## METHODS

### Cell lines

MDA-MB-468 cells (received from Myles Brown, Dana-Farber Cancer Institute) and STAT3 luciferase reporter cells [27] were maintained in DMEM with 10% fetal bovine serum. SUM159PT cells (received from Kornelia Polyak, Dana-Farber Cancer Institute) were maintained as previously described [42]. SK-BR-3 cells (received from Lyndsay Harris, Dana-Farber Cancer Institute) were maintained in RPMI with 10% fetal bovine serum. Cell lines were authenticated on January 29, 2013, by STR DNA profiling (Genetica DNA Laboratories, Cincinnati, OH) and passaged for less than 6 months after thawing. All cells were maintained in a humidified incubator at 37 °C with 5% CO _2_.

### Cytokine stimulations

Cells were stimulated with 10 ng/mL leukemia inhibitor factor (EMD Millipore, Billerica, MA), 10 ng/mL oncostatin-M (R&D Systems), 10 ng/mL interleukin-6 (Peprotech, Rocky Hill, NJ), 100 ng/mL prolactin (R&D Systems, Minneapolis, MN), 10 ng/mL interferon γ (R&D Systems), or 1 μg/mL recombinant human progranulin (PGRN; R&D Systems). Cells were stimulated for 15 minutes for protein analyses, 90 minutes for mRNA analyses, 30 minutes for ChIP, and 6 h for luciferase reporter assays.

### Plasmids

Human GRN/pcDNA3.1v5HIS-TOPO was obtained from Babykumari Chitramuthu (McGill University, Montreal, Canada). Mouse GRN/pCMV6-AC-GFP was purchased from Origene (Rockville, MD). A pCMV6AC-GFP vector control was obtained from Sophia Adamia (Dana-Farber Cancer Institute). STAT3C/pRcCMV was obtained from Jacqueline Bromberg (Memorial Sloan-Kettering Cancer Center, New York, NY).

### Immunoprecipitation

Immunoprecipitations were performed as previously described [27]. Briefly, cells (5 × 10^6^) were lysed on ice for 15 minutes in lysis buffer [50mM Tris (pH 7.5), 150 mM NaCl, 0.5% NP40] with phosphatase and complete protease inhibitors (Roche, Indianapolis, IN). Five percent of the total protein lysate (approximately 12.5 μg of 250 μg) was saved as an immunoprecipitation input loading control, then the remaining lysate was divided into equal volumes for overnight incubation at 4 °C with 1 μg of an antibody to STAT3 (sc-482, Santa Cruz Biotechnology, Inc., Dallas, TX), GRN (40-3400, Invitrogen, Grand Island, NY), or isotype control IgG (Caltag, Burlingame, CA). The lysate-antibody mixture was incubated for 4 hours at 4 °C with protein A/G PLUS agarose beads (Santa Cruz) blocked with 1% bovine serum albumin (Sigma-Aldrich) for 1 hour at 4 °C. The antibody-bead mixture was washed 3 times in lysis buffer, and eluted in sample buffer with10% β-mercaptoethanol.

### Mass spectrometry

#### LC-MS/MS sample preparation

Agarose bead-bound complexes were centrifuged at 14,000*g* for 5 minutes, then re-suspended in 200 μL of 10% acetic acid and 0.1% trifluoroacetic acid (TFA) (pH ~2) and incubated at 65°C for 10 minutes. Elution buffer was collected and dried using a SpeedVac. Eluted complexes were reconstituted in 5 mM TCEP (Sigma-Aldrich, Saint Louis, MO) and 25 mM ammonium bicarbonate (Sigma-Aldrich) pH 8.0, then incubated at 37 °C for 30 minutes. Iodoacetoamide (Sigma-Aldrich) was added to 25 mM and the samples incubated at 37 °C for 1 hour. The same amount of TCEP was added and the samples incubated at room temperature for 1 hour. Digestion was carried out by sequencing grade trypsin (1 μg per sample, Promega, Madison, WI) at 37 °C for 24 hours as described [43] and quenched by addition of formic acid (FA, Thermo Fisher Scientific Inc., Fair Lawn, NJ) to 0.5%.

#### LC-MS/MS Data Acquisition

Two μL (5% of sample) were injected directly into a 35 cm long in-house packed column with a pulled outlet tip. The column was made from a 40 cm long, 75 μm internal diameter fused silica capillary (Molex, Lisle, IL) laser-pulled to a 5 μM tip and packed with 3 μM Magic C18 AQ beads. Samples were loaded in 98% Solvent A (0.1% aqueous FA) for 18 minutes and separated over a 40 minute linear gradient from 2 to 37% of Solvent B (0.1% FA in acetonitrile, Thermo Fisher Scientific) at a flow rate of 200 nL/min. The column was cleaned and equilibrated by a ramp of Solvent B to 95% for 7 minutes and a return to 2% for 8 minutes. Samples were electrosprayed from the pulled tip using a 2.8 kV potential applied at the column inlet.

MS1 scan range was set at 350-1600 Th with resolution 60,000 in the Orbitrap. MS2 scans were conducted in the linear ion trap on the 9 highest intensity ions (‘Top 9’ method) above the minimum signal threshold of 500. CID fragmentation was set with a 2.00 Th isolation window, normalized collision energy of 35.0, activation Q at 0.250, and 30.000 ms activation time. Dynamic exclusion duration was set to 40 seconds with a repeat count of 2 and repeat duration of 15 seconds. Samples were assayed in triplicate.

#### LC-MS/MS Data Analysis

Proteome Discoverer 1.3 (Thermo Fisher Scientific) with combined MASCOT and SEQUEST search was used to identify proteins and relative protein quantities were assessed by peptide-spectral match (PSM) counts (Proteome Discoverer) and peptide peak area based label-free quantitation techniques using MaxQuant 1.4.1.2 [44, 45]. The November 2012 *H. Sapiens* UniProt database including isoforms (74,390 proteins + 47 contaminants) was used for searches. Proteome Discoverer protein identifications were filtered using FDR < 1% for peptide identifications and then by removing proteins identified by a single peptide and by fewer than 6 PSMs. For all searches, the maximal precursor mass error was set to 20 ppm and the fragment mass error was set to 0.6 Da. Oxidation of methionine and deamidation of asparagine and glutamine were selected as dynamic modifications, and carbamidomethylation of cysteine was selected as a static modification. Up to 2 missed tryptic cleavages and fully tryptic peptide sequences were allowed.

### RNA interference

Cells were transfected with 10 nM of siRNA targeting granulin (acrogranin; sc-39621 pool, sc-39621B 5′-GCUUCCAAAGAUCAGGUAATT-3′, sc-39621C 5′-GGACAGUACUGAAGACUCUTT-3′, Santa Cruz), STAT3 (D-003544-03-0010), or non-targeting siControl (D-001210-03-20, Dharmacon Fisher Scientific, Pittsburgh, PA), using Lipofectamine RNAiMAX (Invitrogen) for forty-eight hours.

### Immunoblot analyses

Cells (5 × 10^5^) were lysed on ice for 15 minutes in EBC lysis buffer [50 mM Tris (pH 8.0), 250 mM NaCl, 0.5% NP40] with phosphatase and complete protease inhibitors (Roche). Blots were probed with antibodies to STAT3 (sc-482), hsp90 (sc-13119) from Santa Cruz; phospho-STAT3 (Tyr705) (9131), PARP (9542S), and His-Tag (2365) from Cell Signaling Technology, Inc. (Danvers, MA); GRN (PCDGF, 40-3400) from Invitrogen; FLAG M2 (F1804) and tubulin (T5168) from Sigma-Aldrich; GRN (granulins, PA5-27275) from Thermo Fisher Scientific; turboGFP (TA150041) from Origene (Rockville, MD); and phospho-STAT3 (Ser727) previously generated in rabbits [46]. Immunoblots were imaged on an ImageQuant LAS 4000 and quantified with ImageJ (National Institutes of Health, Bethesda, MD). Nuclear and cytoplasmic fractionations were performed according to manufacturer's instructions (Active Motif Nuclear Extract Kit Cat. No. 40010, Carlsbad, CA).

### Luciferase reporter gene assays

Cells (5 × 10^4^) were transfected with 1 μg of a STAT3-dependent reporter (m67-luc (J. Bromberg, Memorial Sloan-Kettering Cancer Center, New York, NY) or B-luc [47]) and 0.1 μg of *Renilla* luciferase transfection control phRL TK-luc (Promega) using Lipofectamine 2000 (Invitrogen). Twenty-four hours after transfection, cells were stimulated with cytokine or lysed directly and quantitated using dual-luciferase reagents (Promega) on a Luminoskan Ascent luminometer (ThermoLab Systems, Helsinki, Finland). STAT3-dependent luciferase production was normalized to *Renilla* luciferase.

STAT3 luciferase reporter cells stimulated with IL-6 and PGRN for 6 h were analyzed by Bright-Glo luciferase assay (Promega) on a Luminoskan Ascent luminometer (ThermoLab Systems).

### mRNA analyses

Total RNA was extracted from cells (5 × 10^5^) using RNeasy Mini kits (Qiagen, Valencia, CA) and reverse transcribed with TaqMan kits (Applied Biosystems, Foster City, CA). Quantitative PCR was performed in triplicate using SYBR select master mix (Applied Biosystems) on a 7300 or 7500 real-time PCR system (Applied Biosystems). Specificity of amplification was confirmed by melt curve analysis. Cycle threshold (C ) values for target isoforms were normalized to the indicated endogenous reference gene. Primer sequences (Supplemental Table 1) were designed from UCSC genome browser reference transcript sequences using Primer3.

### Chromatin immunoprecipitation

ChIP was performed as previously described [48]. Briefly, cells (1 × 10^7^) were fixed in 1% formaldehyde for 10 minutes, sonicated in 15 second pulses using a Fisher Scientific Sonic Dismembranator Model 500 PDQ on setting 15, and lysates immunoprecipitated overnight at 4°C with an antibody for STAT3 (sc-482) from Santa Cruz. Quantitative PCR was performed using the indicated primers (Supplemental Table 1), and signal detected is expressed as % of input.

### Quantitation of viable cell number

Cells (1 × 10^4^) were transfected with siRNA targeting GRN, STAT3, or a non-targeting control in triplicate. Seventy-two hours later, viable cell number was quantitated by measuring intracellular ATP using Cell Titer-Glo (Promega) on a Luminoskan Ascent luminometer.

### Clonogenic assays

SUM159PT cells (5 × 10^4^) were transfected with siRNA targeting GRN, STAT3, or a non-targeting control for 24 hours, then trypsinized and seeded at 1000 cells/well. After 7 days, colonies were washed in PBS, then fixed and stained in 0.5% crystal violet and 6% glutaraldehyde. Stains were solubilized in 10% acetic acid and measured at OD 590nm on a Spectramax M3 (Molecular Devices, Sunnyvale, CA).

### Migration assays

SUM159PT cells (1 × 10^6^) were transfected with siRNA targeting GRN, STAT3, or a non-targeting control for 48 hours, then the confluent monolayer was disrupted with a linear scratch made with a sterile P200 pipette tip. Cells were washed in PBS, incubated in serum-free media to prevent proliferation, and imaged at 24 hour intervals.

### Immunofluorescence assay

Cells grown on glass cover slips were fixed with 4% paraformaldehyde/PBS for 20 min, permeabilized with 0.5% Triton X-100/H_2_O for 30 min, and blocked in 5% bovine serum albumin/PBS for 1 hour at room temperature. Cells were costained with a mouse antiSTAT3 antibody (1:500 dilution, 9139; Cell Signaling) and a rabbit anti-GRN antibody (1:100 dilution, 40-3400; Invitrogen) overnight at 4C, followed by Alexa Fluor 568-conjugated goat anti-mouse secondary antibody (1:500 dilution, A-11004; Life Technologies) and Alexa Fluor 488-conjugated donkey anti-rabbit secondary antibody (1:500 dilution, A-21206; Life Technologies) for 1 hour. Cover slips were mounted onto glass slides using DAPI-containing Vectashield mounting medium (H-1200; Vector Laboratories, Inc., Burlingame, CA). Images were acquired using an Olympus BX51 fluorescence microscope with attached DP71 camera and DP Manager Software. Three microscopic fields/slide were photographed (40x).

### Computational analyses

Level 3 mRNA expression (Agilent 244k array) and clinical parameters were downloaded from The Cancer Genome Atlas (TCGA) breast carcinoma dataset on October 18, 2013. A second set of previously published gene expression data for 129 breast tumors profiled using Affymetrix U133p2.0 microarrays were downloaded from the Gene Expression Omnibus (GSE5460). PhosphoSTAT3 immunohistochemistry for the corresponding tissue microarrays were previously reported [44]. Correlation analysis between the mRNA levels of GRN and genes in STAT3 expression signatures were conducted with GraphPad Prism 6 Software (La Jolla, CA). The significance of the correlation was calculated using Pearson's correlation. Kaplan-Meier survival analyses were performed with GraphPad Prism 6 Software. Differences in observed survival between groups were tested for significance using the log-rank (Mantel-Cox) test.

### Statistical analyses

Two-tailed T tests for paired samples were performed with Graphpad Prism 6 (La Jolla, CA). Values of p < 0.05 were considered significant (*, *p* < 0.05; **, *p* < 0.01; and ***, *p* < 0.001). Data are presented as mean ± SD for the indicated number of independent experiments (Fig. 2B, 2D top, 3D, 3E, 4C, 4E, 5A, 5B; Supp. Fig. S2B, S6C, S7C&D), otherwise as mean ± SEM for one representative replicate.

## SUPPLEMENTARY MATERIAL, FIGURES AND TABLE


