# Supplementary material for: Granulin, a novel STAT3-interacting protein, enhances STAT3 transcriptional function and correlates with poorer prognosis in breast cancer
Source: Genes Cancer. 2015 Mar;6(3-4):153–68. doi: 10.18632/genesandcancer.58 (PMC4426952; doi:10.18632/genesandcancer.58)
Supplement: Supplementary file 1 [file ganc-06-153-s001.pdf]

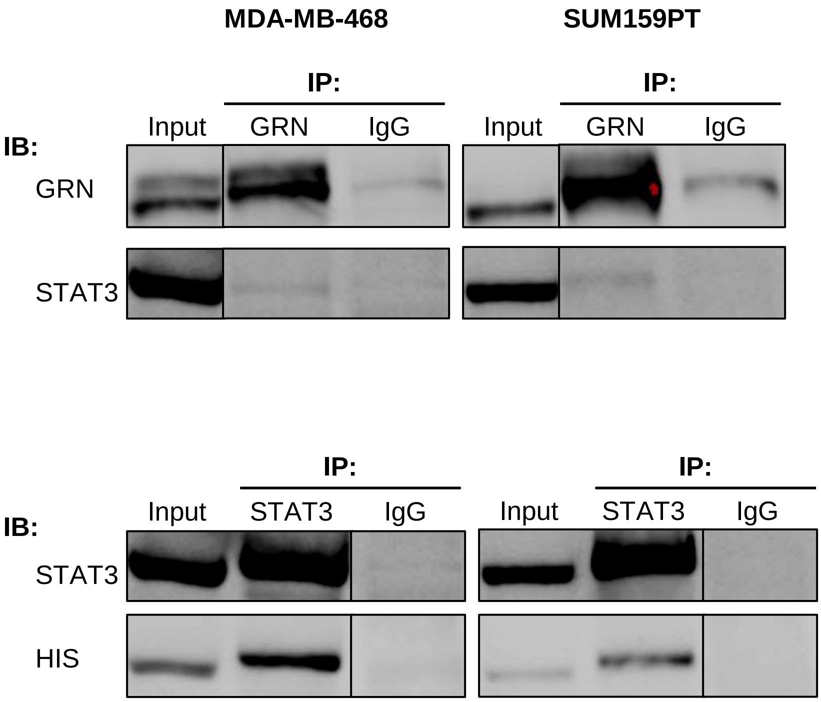

Fig. S1. Reciprocal co-immunoprecipitation of STAT3-GRN complex in TNBC cells.

STAT3-GRN interaction in MDA-MB-468 and SUM159PT cells expressing HIS-tagged GRN were analyzed by immunoprecipitation using antibodies for GRN, STAT3, or a non-specific immunoglobulin G (IgG), followed by immunoblots with the indicated antibodies. Input indicates 5% of pre-immunoprecipitated samples.

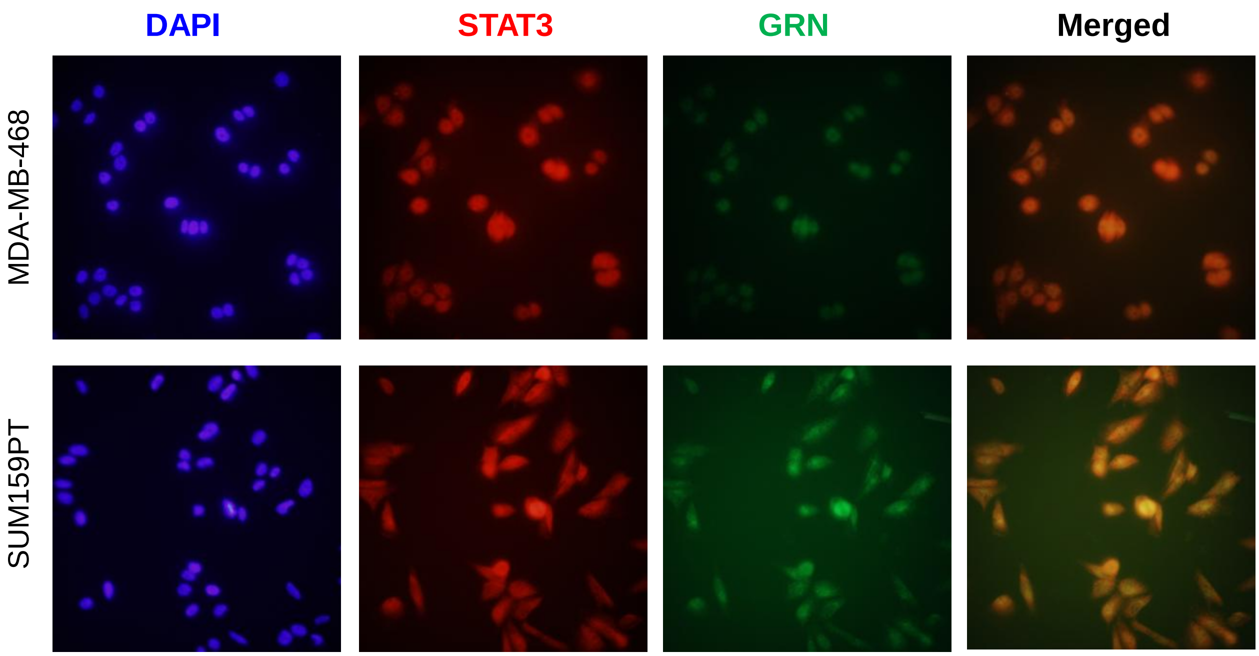

Fig. S2. Immunofluorescent staining showing the colocalization of STAT3 and GRN in TNBC cell lines MDA-MB-468 and SUM159PT. Nuclei were counterstained with DAPI (blue).

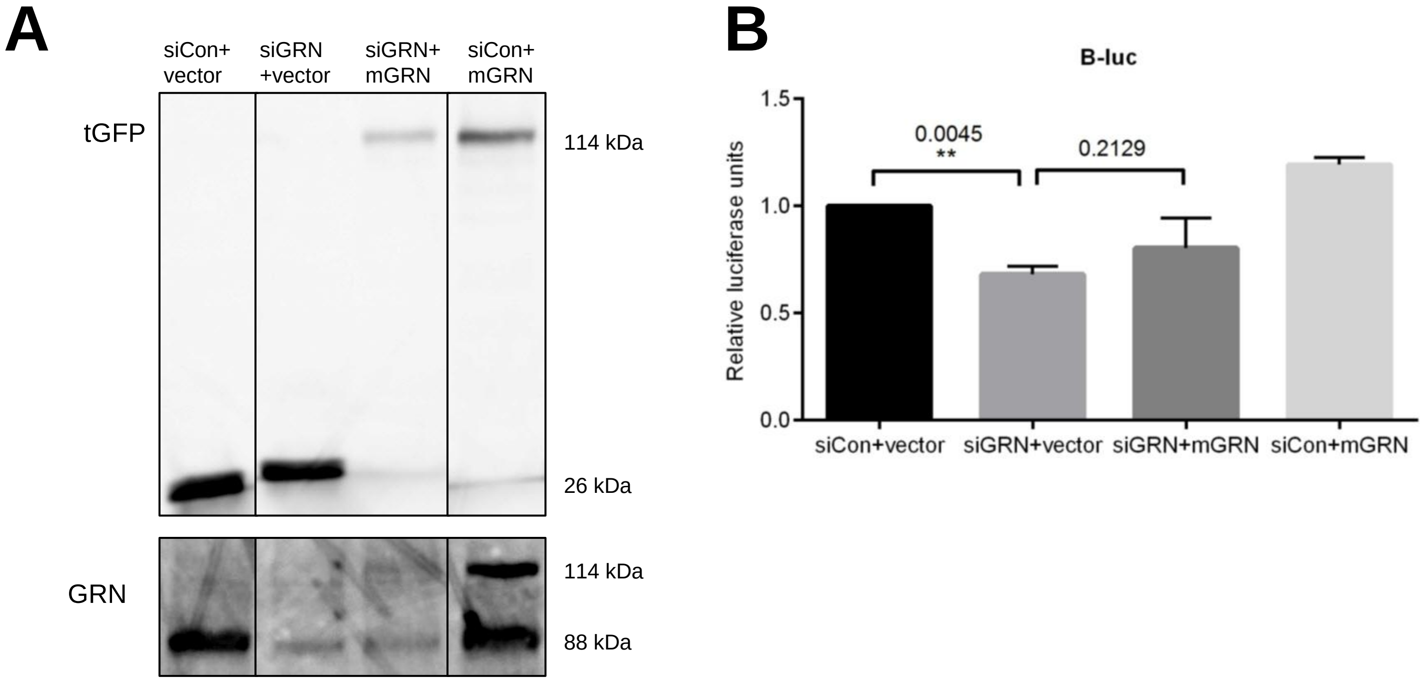

Fig. S3. SUM159PT cells cotransfected with siRNA targeting GRN and a turboGFP (tGFP)-tagged expression construct for mouse GRN (mGRN) were analyzed by **(A)** immunoblot (GRN antibody recognizes both human and mouse GRN protein) and by **(B)** STAT3-dependent luciferase reporter assay (N = 3). siCon, non-targeting control siRNA. mGRN has a larger molecular weight on immunoblot due to addition of tGFP tag, 26 kDa.

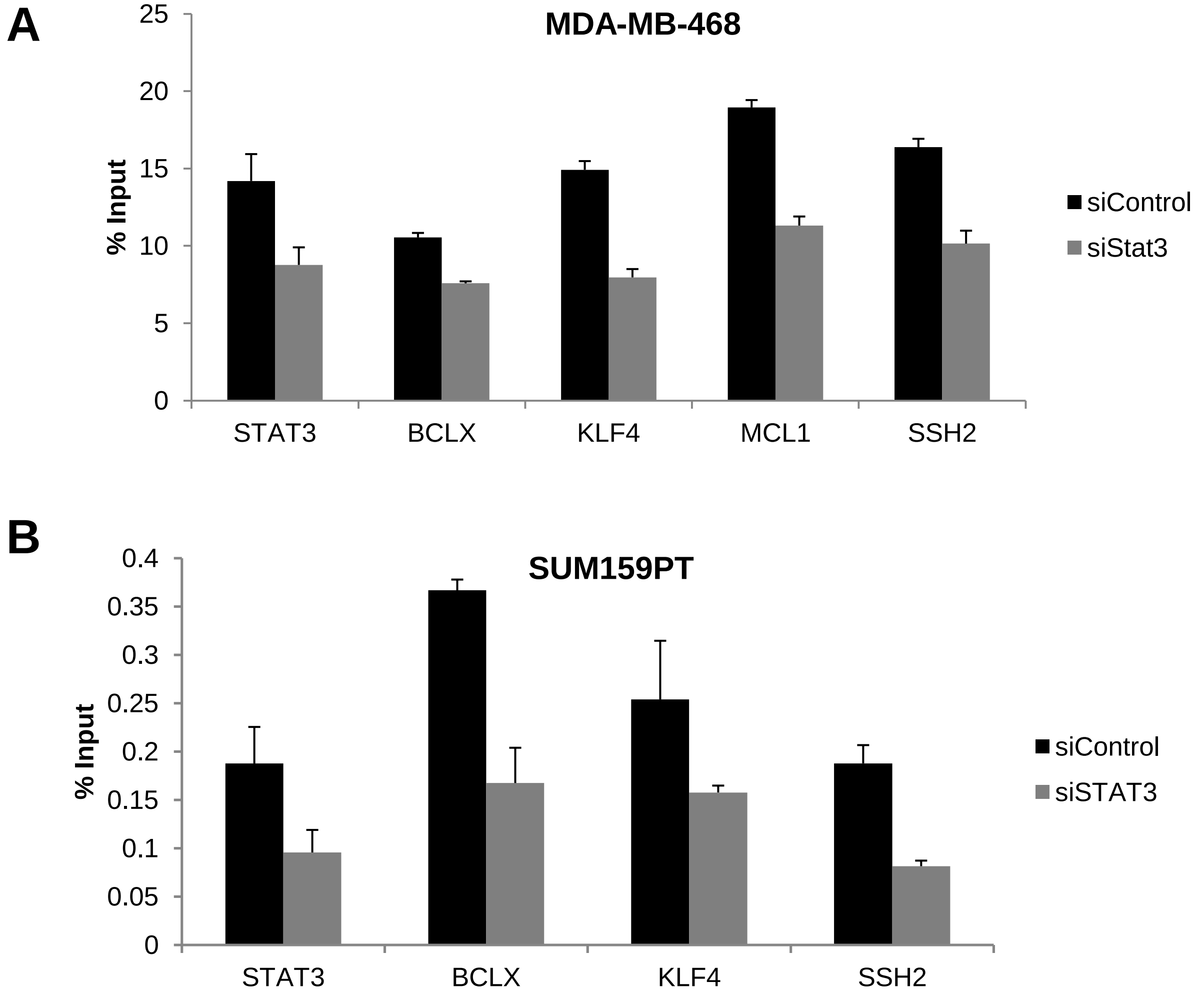

Fig. S4. Validation of STAT3-bound genes in TNBC cells. Chromatin immunoprecipitation with an antibody to STAT3 in **(A)** MDA-MB-468 cells and **(B)** SUM159PT cells transfected with siRNA targeting STAT3 or a non-targeting control. Data expressed as % input.

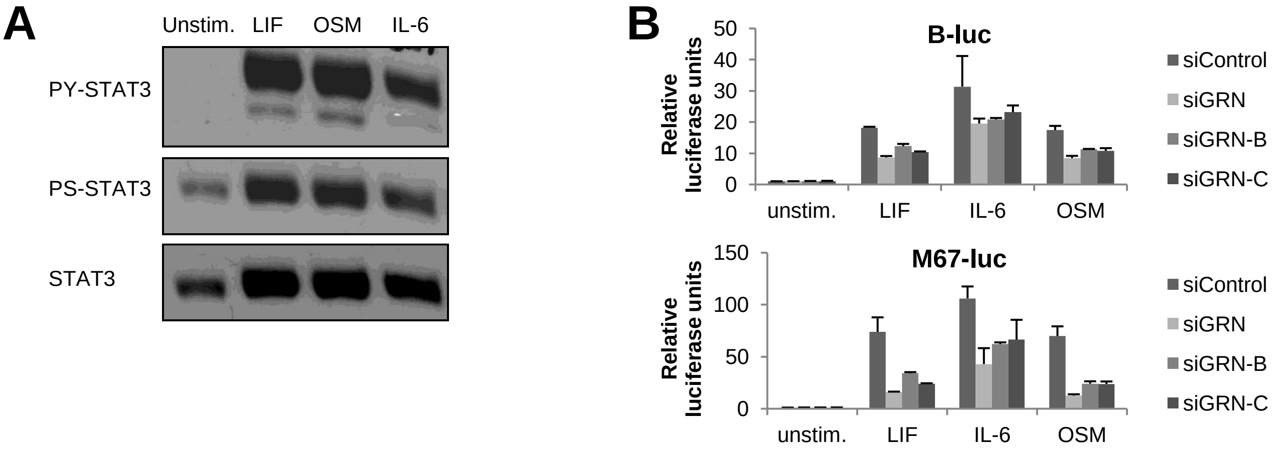

Fig. S5. **(A)** SK-BR-3 cells stimulated with LIF, oncostatin M (OSM), or interleukin-6 (IL-6) were analyzed by immunoblot for levels of tyrosine-phosphorylated (PY-STAT3), serine-phosphorylated (PS-STAT3), and total STAT3. **(B)** SKBR3 cells transfected with siRNA targeting GRN then stimulated with LIF, OSM, or IL-6 were analyzed by luciferase reporter assay for STAT3-driven transcriptional activity.

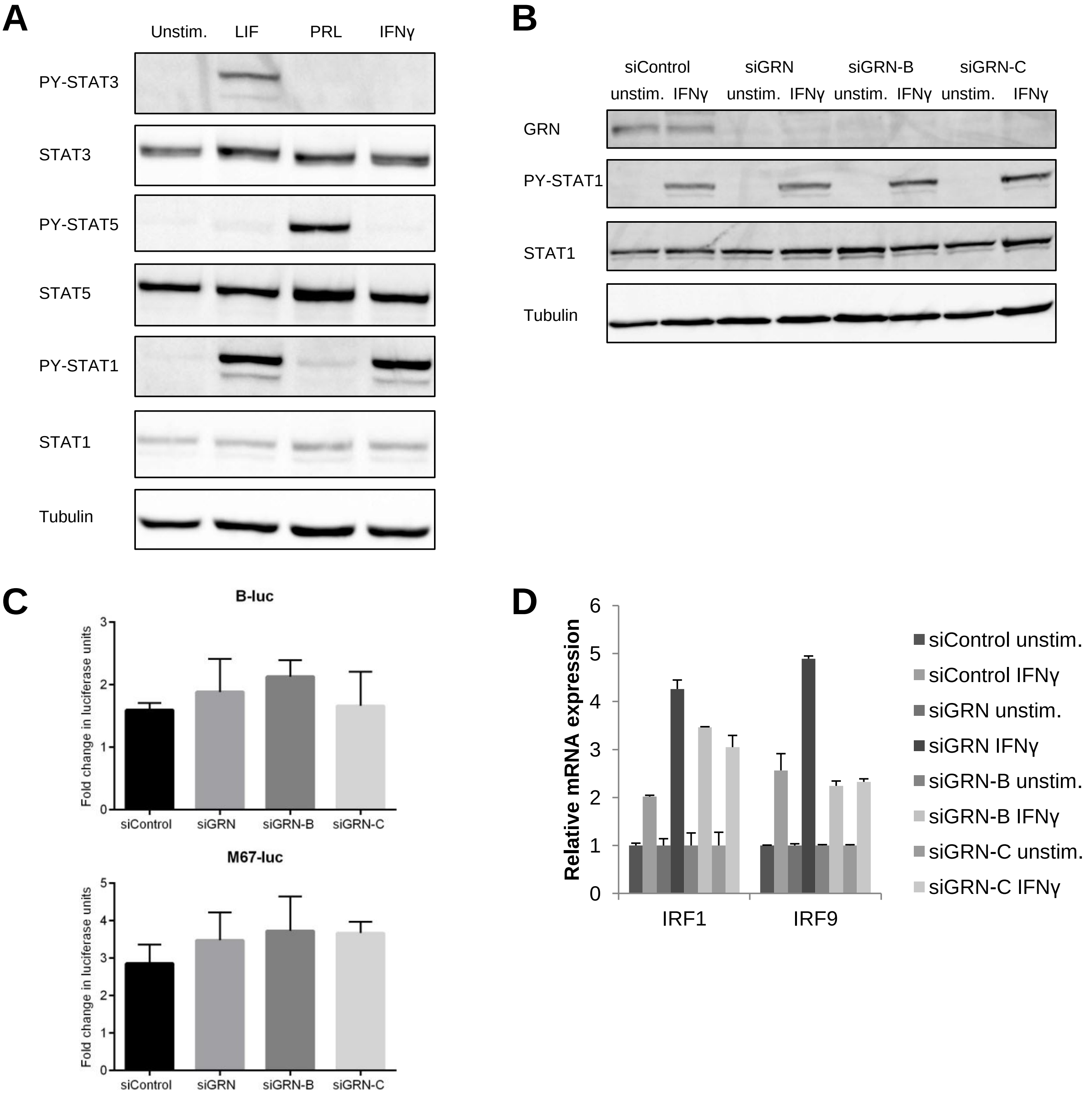

Fig. S6. (A) SK-BR-3 cells stimulated with LIF, prolactin (PRL), or interferon γ (IFNγ) were analyzed by immunoblot for levels of tyrosine-phosphorylated (PY) and total STAT proteins. SK-BR-3 cells transfected with siRNA targeting GRN (pool siGRN and individual constructs siGRN-B and siGRN-C) then stimulated with IFN γ were analyzed by **(B)** immunoblot, **(C)** luciferase reporter assay for STAT1-dependent transcriptional activity (N = 3) and **(D)** qRT-PCR for expression of endogenous STAT1 target genes (normalized to HPRT; representative of N = 3).

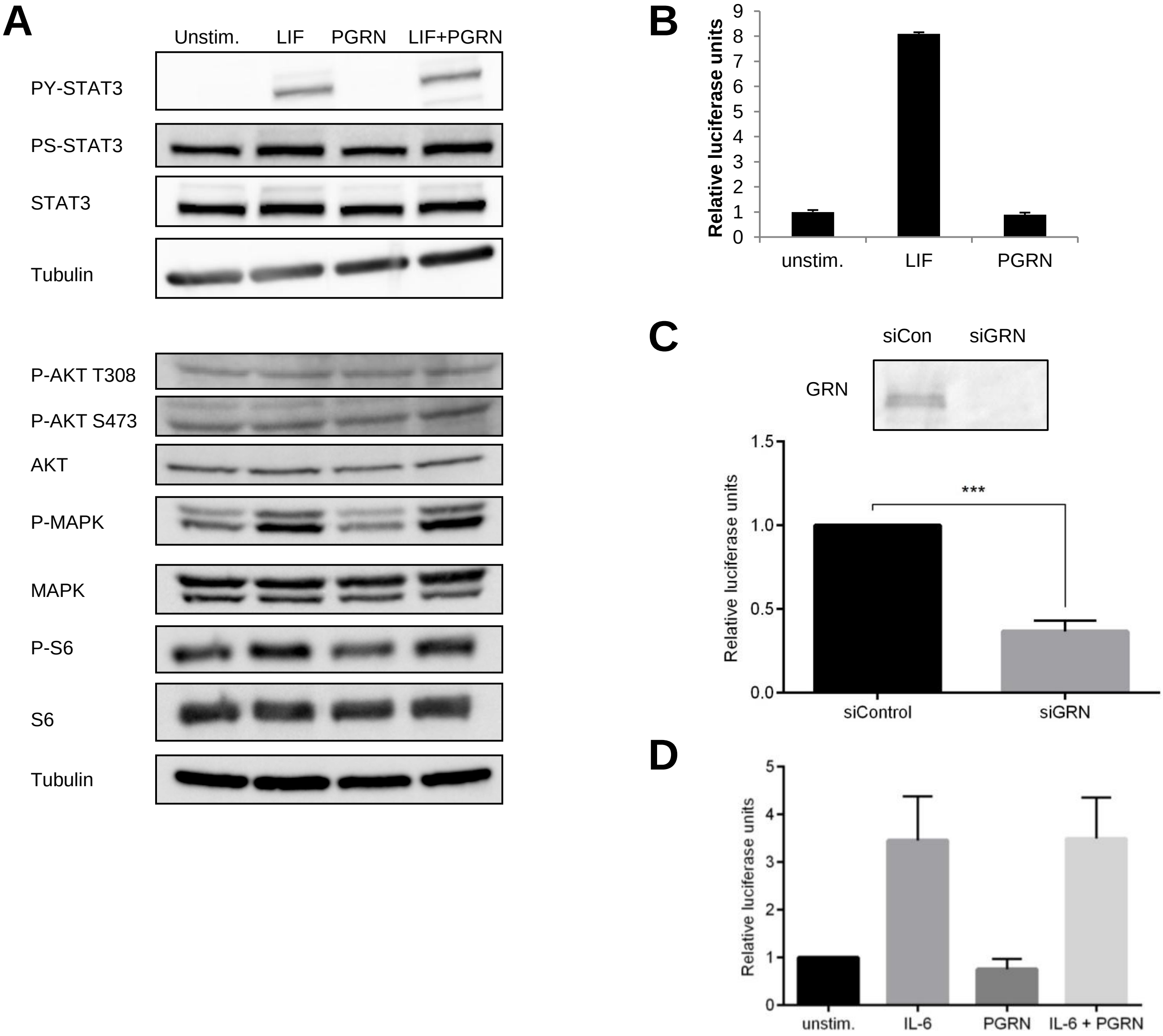

Fig. S7. Extracellular granulin does not activate STAT3 tyrosine phosphorylation or transcriptional activity in breast cancer cells.

SK-BR-3 cells stimulated with LIF or recombinant human progranulin (PGRN) were analyzed by **(A)** immunoblot and **(B)** STAT3-specific luciferase reporter assay. **(C)** STAT3 luciferase reporter cells transfected with siRNA targeting GRN for 48 hours were stimulated with IL-6 for STAT3-specific activation then analyzed by Bright-Glo Luciferase Assay (Promega) on a Luminoskan Ascent luminometer (N = 4). Immunoblot verifies depletion of GRN protein levels. **(D)** STAT3 luciferase reporter cells stimulated with IL-6 and PGRN, alone and in combination, were analyzed by Bright-Glo for luciferase production (N = 3).

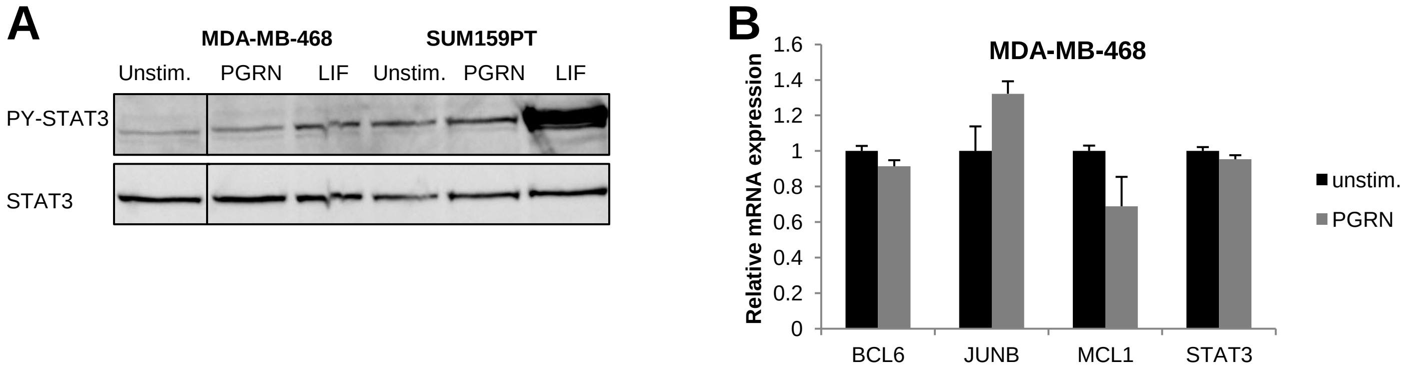

Fig. S8. (A) MDA-MB-468 cells stimulated with PGRN were analyzed by immunoblot for levels of tyrosine-phosphorylated (PY-STAT3) and total STAT3. LIF serves as a positive control for induction of STAT3 activity. **(B)** MDA-MB-468 cells stimulated with PGRN were analyzed by qRT-PCR for mRNA levels of the indicated STAT3 target genes.

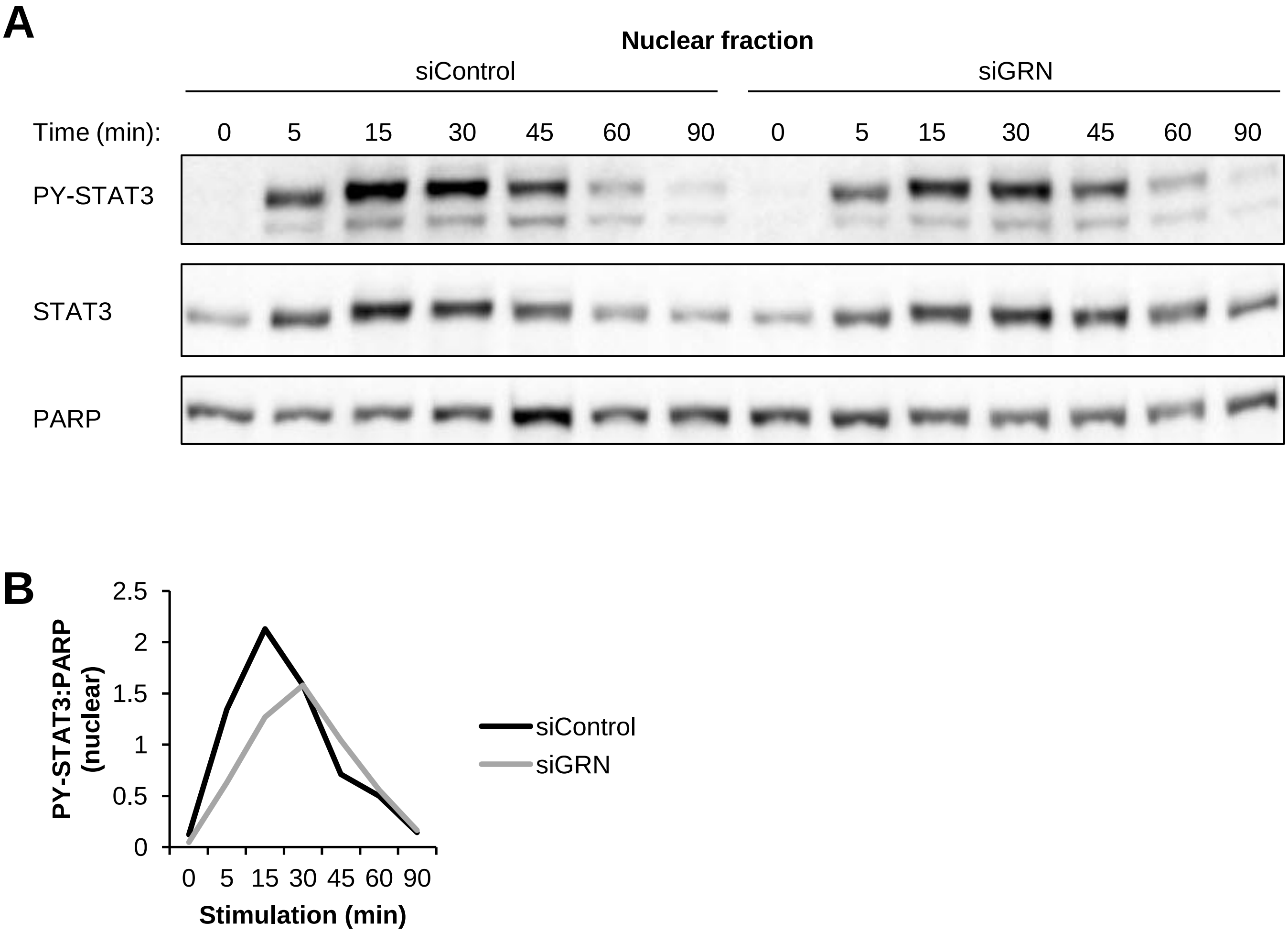

Fig. S9. Silencing GRN reduces cytokine-stimulated nuclear accumulation of STAT3.

(A) SK-BR-3 cells were transfected with siRNA targeting GRN or a non-targeting control for 48 hours, then stimulated with LIF for the indicated times. Cells were harvested and analyzed by immunoblot for levels of tyrosine-phosphorylated (PY-STAT3) and total STAT3 in nuclear fractions. PARP serves as a nuclear fraction loading control. **(B)** Immunoblot band intensities plotted as ratios of PY-STAT3 to PARP.

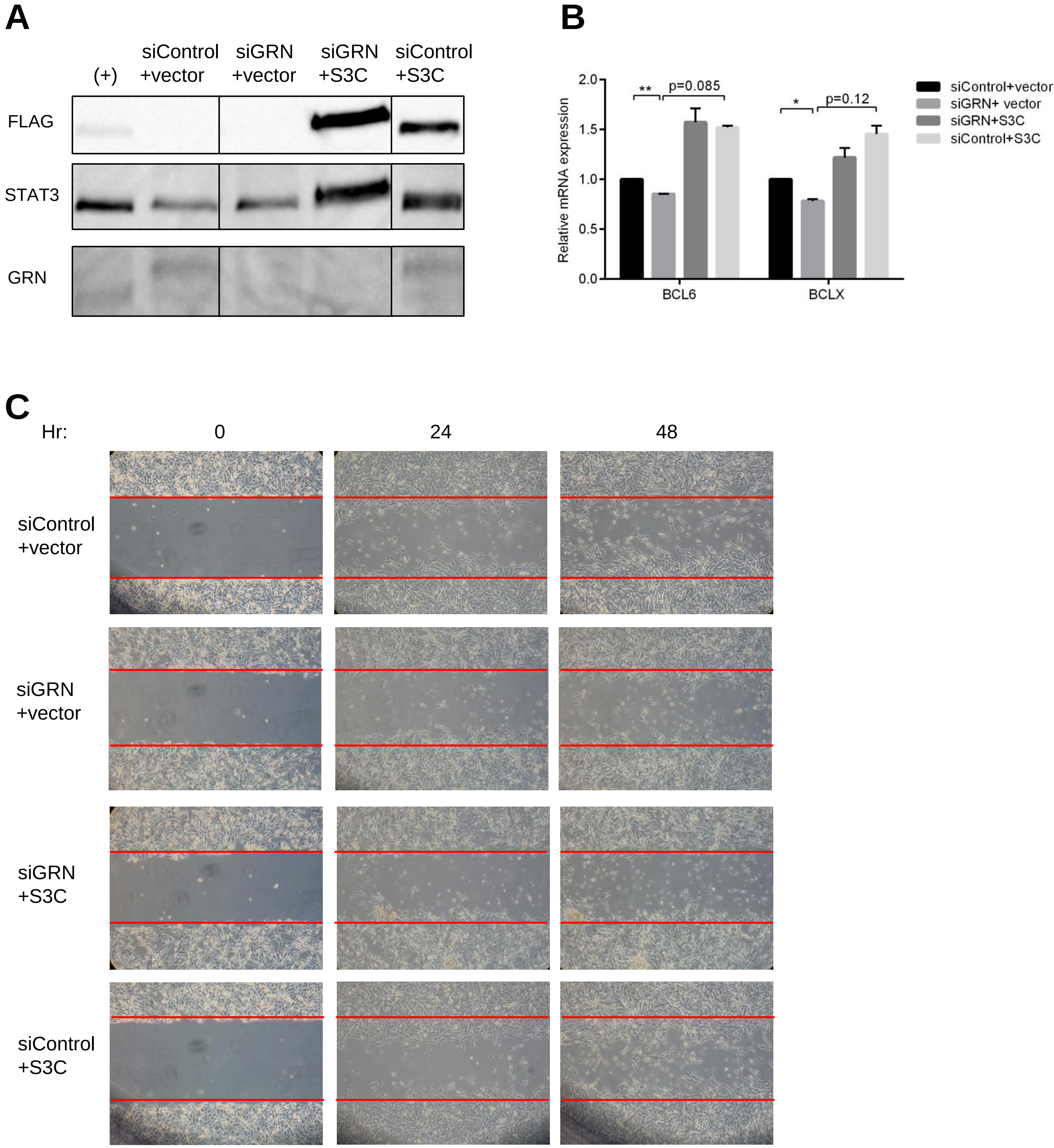

Fig. S10. SUM159PT cells transfected with siRNA targeting GRN and a FLAG-tagged expression construct for constitutively active STAT3 (S3C) were analyzed by **(A)** immunoblot, **(B)** qRT-PCR for expression of the indicated STAT3 target genes normalized to HPRT (N = 2), and **(C)** wound healing assay.

A

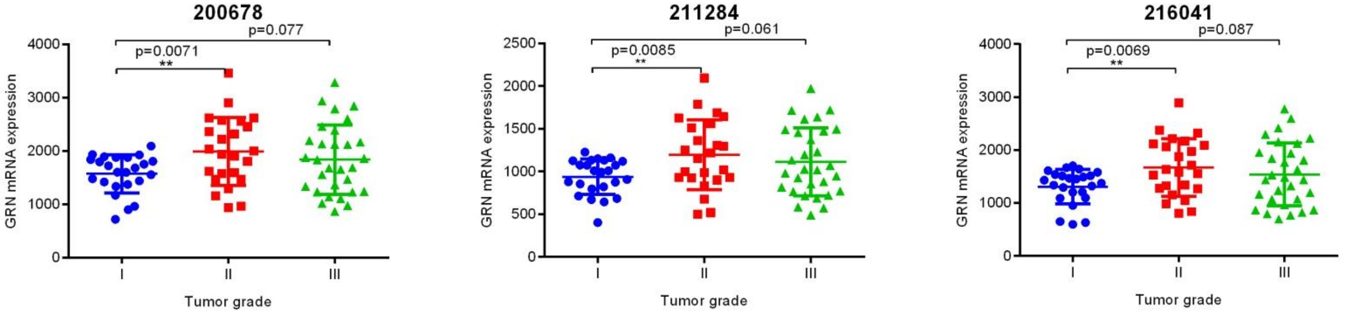

Tumor grade	# tumors
I	24
II	24
III	30

B

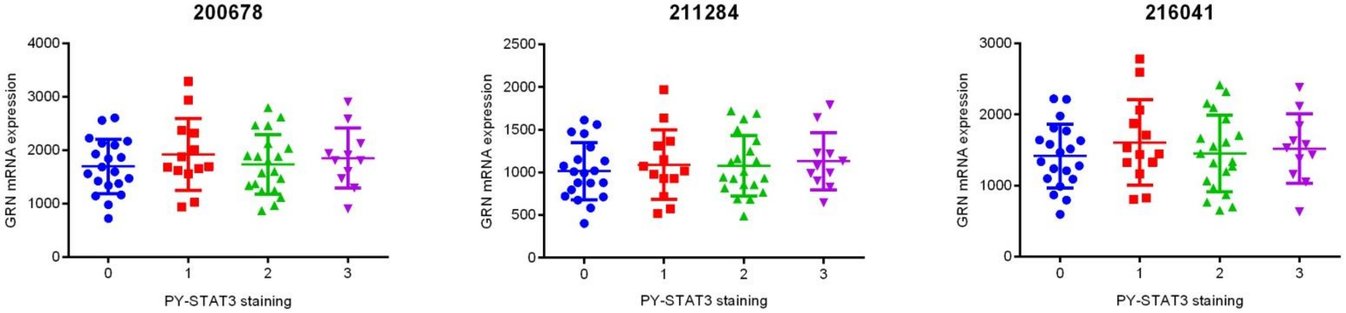

PY-STAT3 staining	# tumors
0	20
1	13
2	20
3	11

Fig. S11. GRN expression is positively correlated with **(A)** tumor grade but not with **(B)** tyrosine-phosphorylated STAT3 (PY-STAT3) histologic staining (scores of 0 to 3+) in breast tumors. 200678, 211284, and 216041 denote three different microarray probes to the GRN transcript.

	Gene	Forward Sequence	Reverse Sequence
hqRT	BATF	CAGCAGTGACTCCAGCTTCA	CCTTCTGTGTCTGCCTCTGTC
	BCL6	CTGCAGATGGAGCATGTTGT	TCTTCACGAGGAGGCTTGAT
	BCLX	GGTATTGGTGAGTCGGATCG	TGCTGCATTGTTCCCATAGA
	CSNK1A1	CCGAGATGACATGGAATCATTA	AAACTTCAACAGGCGTGGAC
	GAPDH	AATCCCATCACCATCTTCCA	TGGACTCCACGACGTACTCA
	GRN	TCTGTAGTCTGAGCGCTACCC	GTTAAGGCCACCCAGCTCAC
	HPRT	GAACGTCTTGCTCGAGATGTG	CCAGCAGGTCAGCAAAGAATT
	IRF1	AAGGGAAATTACCTGAGGACATCAT	CAATTTCTGGCTCCTCCTTACAGCTAA
	IRF9	CCCATCTCCTGGAATGCAC	CATGGCTCTCTTCCCAGAAA
	JUNB	AAATGGAACAGCCCTTCT	TGTAGAGAGAGGCCACCA
	KLF4	TCCCATCTTTCTCCACGTTC	AGTCGCTTCATGTGGGAGAG
	MCL1	GAGACCTTACGACGGGTT	TTTGATGTCCAGTTTCCG
	SSH2	GGCTCATCCACACCAAGAAT	GTTGTCTTCTGGGCGGAGTA
	STAT3	GAGAAGCCAATGGAGATTGC	GACATCCTGAAGGTGCTGCT
hChIP	BATF	TGTCACCAGTGGAAACTCTCAG	TTGGGTATTTCCTGGCACAT
	BCL6	CGGCAGCAACAGCAATAATC	GGAGAGCTGACACCAAGTCC
	BCLX	CTGGGTTCCCTTTCCTTCCA	TCCCAAGCAGCCTGAATCC
	CSNK1A1	CAGCAGATGGGCAGGTTATT	AGGCAGAACCTCAGGCTGTA
	JUNB	TGGACTCCAGGGAAATCATC	AAGCGCGTGTCCTTGTAAAC
	KLF4	GCCAGTAATGACCAGGAGCTA	CAGAGCGGTAGCTAGGACCA
	LPP	AAAAGCTATCCAAGAAAAGCAG	TTTGCTTCATTGTGGTGGTG
	MCL1	TGTTTCTCTGCTTTGCCTCAT	GCGGTATACTCCTTTCCTCCA
	SSH2	AGCCATACCTGGAACCCTCT	ACTCATGTGGCCCAGTCTCT
	STAT3	CCTGATACAGCTCCCTCCTG	GATTCCCGCGTGGTAAGAG

Supplemental Table 1. Primer sequences used for quantitative real-time PCR analysis (hqRT)of mRNA expression and chromatin immunoprecipitation (hChIP) of STAT3 DNA binding.
